# Atypical Unilateral-Onset Guillain-Barré Syndrome Mimicking Acute Stroke: A Case Report

**DOI:** 10.7759/cureus.107026

**Published:** 2026-04-14

**Authors:** Dia Mobaideen, Gihad Osman, Amir Nur, Muhammad Faizan Latif, Mahnoor Tayyib

**Affiliations:** 1 Acute Medicine, Lincoln County Hospital, Lincoln, GBR; 2 Geriatrics, Lincoln County Hospital, Lincoln, GBR; 3 Medicine, Lincoln County Hospital, Lincoln, GBR; 4 Stroke Medicine, Lincoln County Hospital, Lincoln, GBR

**Keywords:** guillain-barré syndrome (gbs), icu management, intravenous immunoglobulin (ivig), nerve conduction study (ncs), stroke

## Abstract

Guillain-Barré syndrome (GBS) is an acute immune-mediated neuropathy that can present with a wide spectrum of neurological deficits, sometimes in atypical forms that complicate early diagnosis. Atypical presentations may mimic other neurological emergencies, contributing to diagnostic delays. We report the case of a 67-year-old man with a history of alcohol misuse who presented with sudden-onset unilateral lower-limb weakness initially suspected to represent an acute stroke. Over several days, his weakness progressed to involve both lower limbs, accompanied by sensory loss, gait ataxia, and diffuse areflexia. Cerebrospinal fluid (CSF) demonstrated albuminocytologic dissociation, and nerve conduction studies (NCS) confirmed a demyelinating polyneuropathy. The patient subsequently developed respiratory compromise requiring intensive care unit (ICU) monitoring and intubation. Intravenous immunoglobulin (IVIG) was initiated, resulting in gradual clinical improvement. This case highlights the importance of comprehensive neurological examination and maintaining a broad differential diagnosis when evaluating acute focal weakness, particularly when initial findings are incomplete or atypical.

## Introduction

Guillain-Barré syndrome (GBS) is the most common cause of acute flaccid paralysis worldwide and is classically characterised by symmetrical ascending weakness, sensory symptoms, and reduced or absent deep tendon reflexes [1]. Although most cases follow this typical pattern, atypical presentations occur in a significant minority and can obscure the diagnosis. Reported variants include unilateral weakness, cranial nerve-predominant disease, pure sensory forms, and paraparetic presentations [2,3].

Stroke is a common and time-critical diagnosis in older adults presenting with sudden neurological deficits. When a patient presents with acute unilateral weakness, stroke is often the leading consideration. However, stroke mimics may account for up to 30% of suspected stroke presentations [4]. Anchoring bias, the tendency to fixate on an initial diagnosis, may delay recognition of alternative conditions such as GBS, particularly when the neurological examination is incomplete.

GBS is most commonly triggered by preceding infections, particularly gastrointestinal or respiratory illnesses. Epidemiological studies estimate that up to two-thirds of patients report a preceding infectious event, with *Campylobacter jejuni* being the most frequently implicated pathogen [2]. The disease encompasses several subtypes, including acute inflammatory demyelinating polyneuropathy (AIDP), acute motor axonal neuropathy (AMAN), and Miller Fisher syndrome, each with distinct clinical and electrophysiological features [2]. Early recognition is critical, as timely immunomodulatory treatment has been shown to reduce disease severity and improve functional outcomes [5].

## Case presentation

A 67‑year‑old man with a history of chronic alcohol misuse presented to the stroke unit with sudden‑onset progressive weakness in his left leg. The weakness began abruptly and worsened over several hours. He denied facial droop, speech disturbance, visual symptoms, or upper‑limb involvement. There was no headache, seizure activity, or loss of consciousness.

On initial examination, he was alert and oriented. Cranial nerves were intact, and upper‑limb strength was normal. The only documented abnormality was left lower‑limb weakness. No reflexes were assessed. Based on the acute unilateral presentation, a working diagnosis of stroke was made.

Over the next four days, his condition deteriorated. Weakness progressed to involve both lower limbs, and he developed worsening gait ataxia and stocking‑like sensory loss. Despite this progression, reflexes continued not to be examined.

On day 5, a comprehensive neurological assessment was performed. The patient disclosed a recent hospitalisation for an acute diarrhoeal illness several days before symptom onset. Examination revealed bilateral lower‑motor‑neuron pattern weakness, glove‑and‑stocking sensory loss, diffuse areflexia, and normal cranial nerve and cerebellar examination.

During the assessment, shallow breathing and reduced respiratory effort were noted, prompting transfer to the intensive care unit (ICU).

Investigations

His blood and cerebrospinal fluid (CSF) results are shown in Table 1.

**Table 1:** Blood and CSF results CSF: cerebrospinal fluid

Test	Result	Normal range	Units
CSF protein	4.3	0.15-0.45	g/L
CSF white cells	1	0-5	cells/µL
CSF lactate	2.5	1.1-2.4	mmol/L
Blood chemistry	Normal	-	-
Full blood count	Normal	-	-

**Figure 1:**
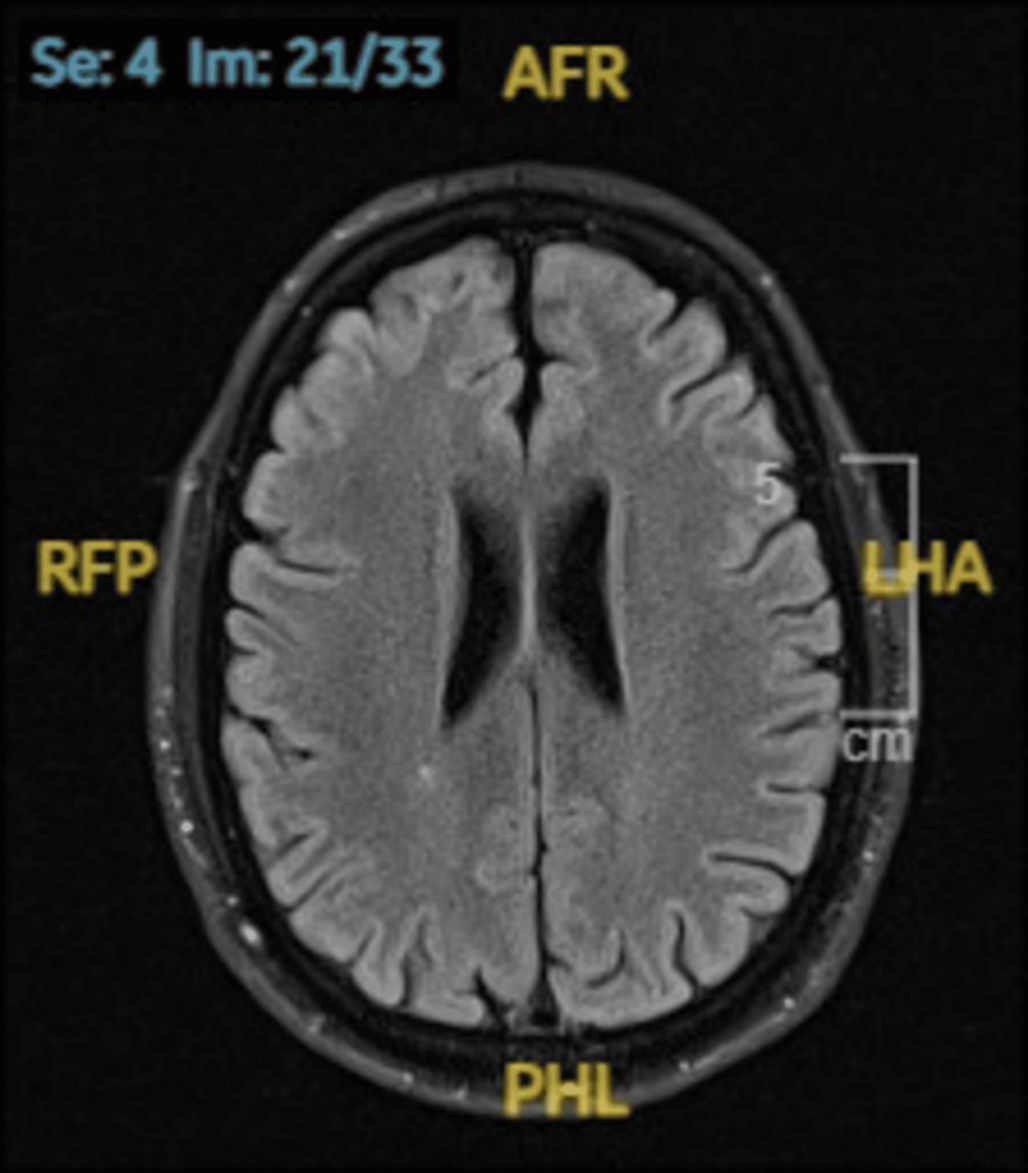
Axial brain FLAIR MRI sequence demonstrating normal cerebral parenchyma with no evidence of acute infarction or focal pathology Image obtained from the hospital radiology PACS and anonymised for publication FLAIR: fluid-attenuated inversion recovery, MRI: magnetic resonance imaging, PACS: Picture Archiving and Communication System

**Figure 2:**
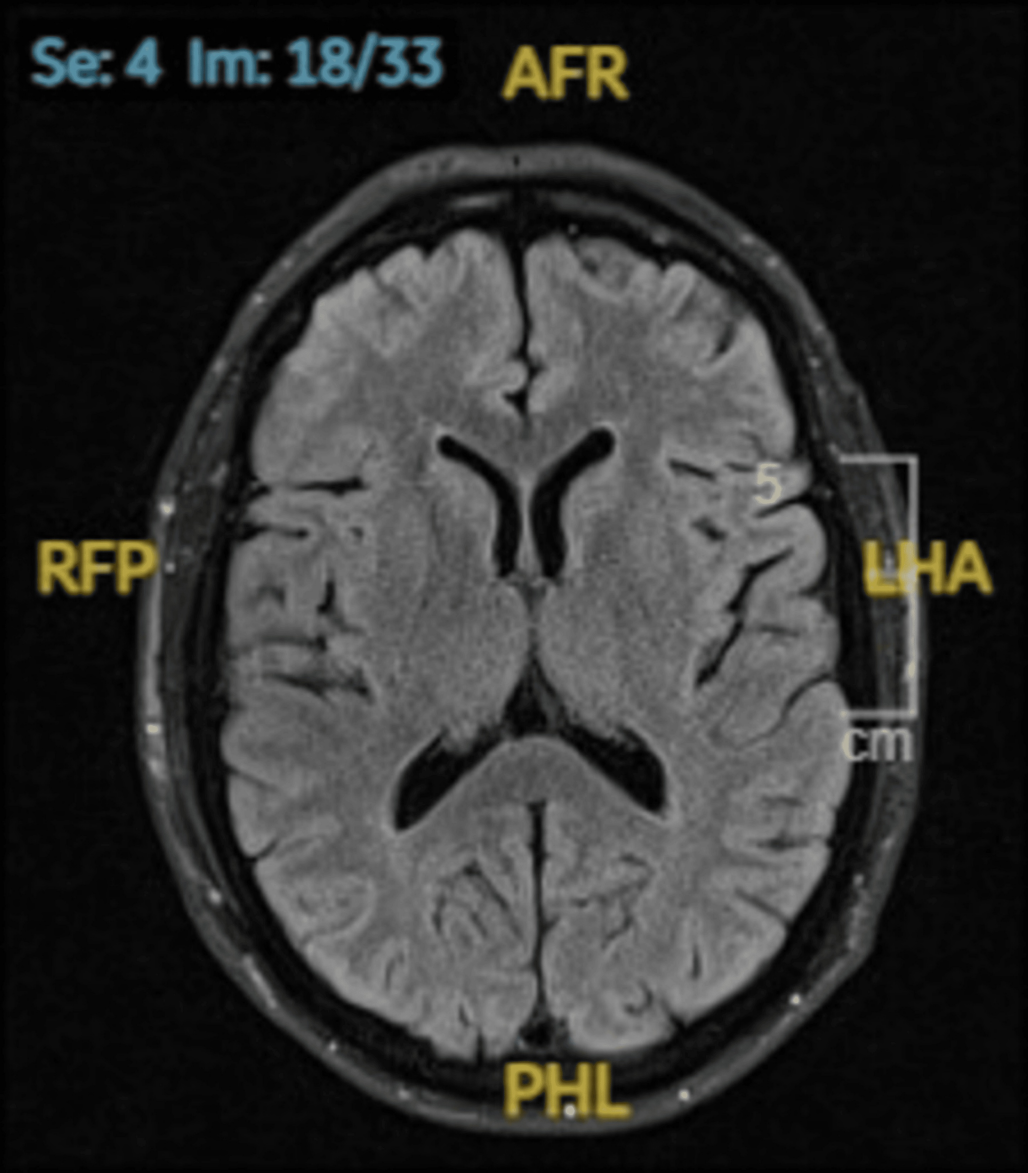
Axial brain FLAIR MRI sequence demonstrating normal cerebral structures with no evidence of acute infarction, haemorrhage, or mass lesion Image exported from the hospital radiology PACS and anonymised for publication FLAIR: fluid-attenuated inversion recovery, MRI: magnetic resonance imaging, PACS: Picture Archiving and Communication System

The patient’s brain magnetic resonance imaging (MRI) was normal, with no evidence of acute infarction (Figure 1 and Figure 2).

The nerve conduction study results, sensory nerve conduction findings, and electromyography findings are shown in Table 2, Table 3, and Table 4, respectively.

**Table 2:** Nerve conduction study results

Nerve	Site	Latency (ms)	Amplitude	Conduction velocity (m/s)	Key finding
Fibular (left)	Ankle	11.8	1.07 mV	-	Markedly prolonged distal latency
Fibular (left)	Popliteal fossa	24.7	0.56 mV	31.0	Reduced amplitude with slowed conduction
Fibular (right)	Ankle	13.3	1.41 mV	-	Prolonged distal latency
Median (right)	Wrist	6.2	1.31 mV	-	Delayed distal motor latency
Tibial (left)	Ankle	4.8	0.75 mV	-	Reduced amplitude
Tibial (right)	Knee	13.9	0.33 mV	44.7	Significant amplitude reduction
Ulnar (right)	Wrist	3.2	3.0 mV	60.0	Within normal limits

**Table 3:** Sensory nerve conduction findings

Nerve	Site	Latency (ms)	Amplitude (µV)	Conduction velocity (m/s)	Finding
Median (right)	Wrist-digit II	NR	NR	NR	Sensory response absent
Median (right)	Wrist-digit III	NR	NR	NR	Sensory response absent
Ulnar (right)	Wrist-digit V	NR	NR	NR	Sensory response absent
Radial (right)	Forearm-snuffbox	1.30	13.2	61.5	Preserved sensory response
Superficial fibular (left)	Ankle	1.73	5.9	52.0	Borderline attenuation
Superficial fibular (right)	Ankle	2.10	5.7	47.6	Borderline attenuation
Sural (left)	Calf	1.43	7.8	59.4	Preserved
Sural (right)	Calf	1.48	8.3	57.4	Preserved

**Table 4:** Electromyography findings

Muscle	Spontaneous activity	Motor unit morphology	Recruitment
Tibialis anterior	None	High amplitude, prolonged duration	Reduced
Gastrocnemius	None	High amplitude, prolonged duration	Reduced
Rectus femoris	None	High amplitude, prolonged duration	Reduced
Biceps	None	High amplitude, prolonged duration	Reduced
First dorsal interosseous	None	High amplitude, prolonged duration	Reduced

The nerve conduction studies demonstrate severe widespread sensorimotor demyelinating large-fibre peripheral neuropathy, characterized by markedly prolonged distal motor latencies, reduced motor amplitudes, temporal dispersion of motor responses, absent sensory responses in the upper limb nerves, and absent tibial F-waves.

These findings are consistent with acute inflammatory demyelinating polyneuropathy (AIDP), the most common subtype of Guillain-Barré syndrome.

Diagnosis

The combination of progressive symmetrical weakness, sensory loss, diffuse areflexia, recent gastrointestinal illness, CSF albuminocytologic dissociation, and nerve conduction studies confirming a demyelinating polyneuropathy was diagnostic of Guillain-Barré syndrome. Although the initial unilateral onset was atypical, such presentations are recognised variants.

Management

The patient was transferred to the neurology service, where intravenous immunoglobulin (IVIG) was initiated at a dose of 0.4 g/kg/day for five consecutive days in accordance with established treatment guidelines two days after diagnosis. Supportive care included respiratory monitoring, physiotherapy, and measures to prevent complications of immobility.

Outcome

Following IVIG therapy, the patient gradually improved. During the acute phase of illness, he developed significant respiratory compromise with reduced peak flow values to less than 1000, necessitating intubation and mechanical ventilation for several days. He was subsequently successfully extubated following improvement in respiratory function. The patient was managed under shared care between the intensive care unit and the neurology team. Over the following weeks, he made a full recovery, regaining normal strength and function, and was transferred to a rehabilitation facility. At follow-up, he remained well with no residual neurological deficits.

**Figure 3:**
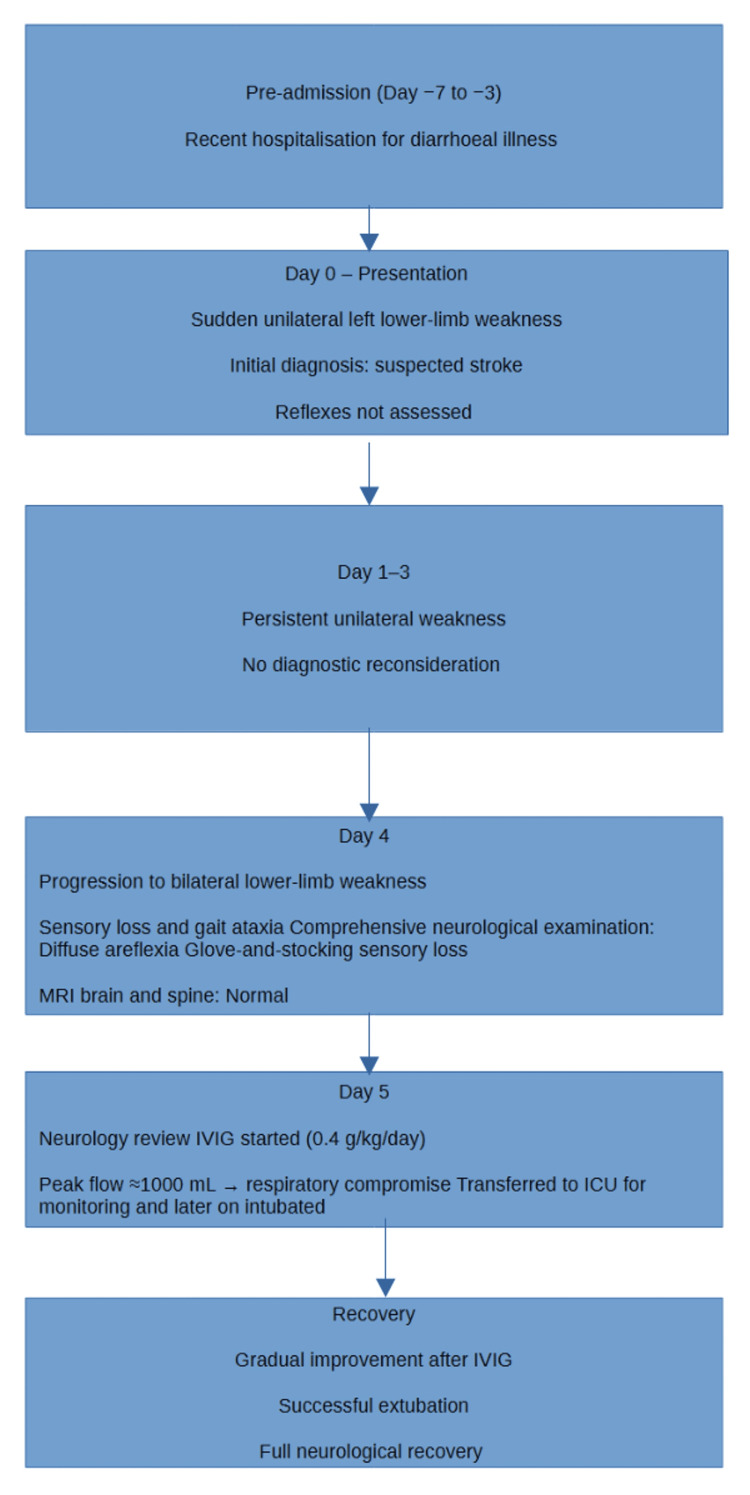
Clinical timeline of disease progression This timeline illustrates the sequence of events from the patient’s preceding gastrointestinal illness through the onset and progression of neurological symptoms, specialist review, initiation of intravenous immunoglobulin therapy, development of respiratory compromise requiring intensive care support, and eventual recovery. ICU: intensive care unit, IVIG: intravenous immunoglobulin, MRI: magnetic resonance imaging Created by the authors using LibreOffice Draw (OpenDocument software) using standard shapes and layout tools without the use of generative artificial intelligence

Figure 3 presents the timeline of the patient’s disease progression.

## Discussion

This case presents several important clinical lessons. First, Guillain-Barré syndrome (GBS) can present atypically, and unilateral weakness, although uncommon, is a recognised variant. Winer describes several clinical variants of GBS, including asymmetrical and focal presentations, which may reflect heterogeneity in immune-mediated nerve injury [3]. Such atypical forms can obscure the diagnosis, particularly early in the disease course, when classical features have not yet fully evolved.

Second, the absence of reflex testing early in the patient’s course contributed significantly to the delay in diagnosis. Reflex examination is simple, rapid, and highly informative. Areflexia is a hallmark of GBS and often precedes significant weakness. Willison et al. emphasise that reduced or absent reflexes are a core diagnostic feature and should prompt consideration of GBS even in the absence of symmetrical weakness [1]. Similarly, Ropper highlights that early clinical recognition remains crucial, as diagnostic investigations may lag behind symptom progression [5].

Third, anchoring bias played a role in this case. Once stroke was suspected, subsequent assessments were interpreted through that lens despite the evolving clinical picture. Progressive bilateral weakness, sensory loss, and ataxia are not typical of stroke. Fokke et al., in their validation of the Brighton criteria, found that the Brighton criteria provide a structured framework for diagnosing GBS [6]. In this patient, the presence of progressive bilateral weakness, diffuse areflexia, and cerebrospinal fluid albuminocytologic dissociation fulfilled level 1 diagnostic certainty when supported by electrodiagnostic evidence of demyelinating neuropathy. The absence of an alternative diagnosis and the monophasic progression further strengthened the diagnostic classification [5]. A comparative summary of distinguishing features between acute stroke and atypical GBS is provided in Table 5 to support clinical differentiation and reduce anchoring bias.

**Table 5:** Key clinical features differentiating acute stroke and atypical Guillain-Barré syndrome Published literature on Guillain-Barré syndrome and stroke mimics [1,4,6]

Feature	Acute stroke	Atypical Guillain-Barré syndrome
Onset	Sudden, maximal at onset	Progressive over hours to days (may appear sudden initially)
Pattern of weakness	Typically unilateral (hemiparesis)	May begin asymmetrically but progresses to bilateral weakness
Progression	Non-progressive or stepwise (depending on stroke type)	Progressive, often ascending pattern
Reflexes	Normal or increased (hyperreflexia)	Reduced or absent (areflexia)
Tone	Increased (spasticity) over time	Reduced (flaccidity)
Sensory symptoms	Variable, often cortical pattern	Glove-and-stocking distribution common
Cranial nerve involvement	Common (e.g., facial droop, dysarthria)	May occur, but often absent early in atypical cases
Coordination	May have cerebellar signs	Ataxia possible due to sensory involvement
Autonomic dysfunction	Less common	Common (e.g., tachycardia, blood pressure fluctuations)
Respiratory involvement	Rare (unless brainstem stroke)	Common in severe cases, may require ventilation

Fourth, this case reinforces that GBS is a medical emergency requiring close monitoring for respiratory compromise. Leonhard et al. outline a structured approach to GBS management, emphasising early recognition of respiratory decline through measures such as vital capacity and peak flow monitoring [7]. In this patient, deterioration in respiratory function necessitated intensive care support and temporary mechanical ventilation, which is required in approximately 20%-30% of GBS cases. Furthermore, early treatment with IVIG or plasma exchange has been shown to shorten recovery time and reduce the need for prolonged mechanical ventilation. Randomised controlled trials demonstrate equivalent efficacy between these modalities, although IVIG is often preferred due to ease of administration and availability. Delayed treatment, particularly in atypical cases, has been associated with increased morbidity, prolonged hospital stay, and slower neurological recovery [6].

Fifth, cerebrospinal fluid (CSF) analysis and nerve conduction studies provide important supportive evidence for diagnosis. The presence of albuminocytologic dissociation, as described by van den Berg et al. [2], reflects increased permeability of the blood-nerve barrier without significant inflammation. Electrodiagnostic studies further help classify GBS subtypes. In this case, findings were consistent with a demyelinating process, supporting a diagnosis of acute inflammatory demyelinating polyneuropathy (AIDP), the most common subtype in Europe and North America.

Finally, the patient’s recent diarrhoeal illness was a key historical feature. Many cases of GBS follow gastrointestinal or respiratory infections, most commonly associated with *Campylobacter jejuni*. Yuki and Hartung describe the role of molecular mimicry in the pathogenesis of GBS [8]. Antibodies generated against infectious agents, particularly *Campylobacter jejuni*, cross-react with gangliosides located on the membranes of peripheral nerves. These gangliosides (such as GM1, GD1a, and GQ1b) share structural similarities with bacterial lipooligosaccharides, leading to an autoimmune response. This immune-mediated attack results in complement activation, macrophage recruitment, and subsequent demyelination or axonal injury, depending on the subtype of GBS [7].

Overall, this case highlights the diagnostic challenges posed by atypical presentations of GBS and underscores the importance of thorough neurological examination, awareness of cognitive bias, and early recognition of disease progression. Recognising neurological conditions that mimic acute stroke is essential in emergency settings, as stroke mimics represent a substantial proportion of suspected stroke presentations and require alternative diagnostic and management strategies [8].

Limitations

This report has several limitations. First, it describes a single patient, limiting generalisability. Second, the patient’s history of chronic alcohol misuse may have confounded early neurological assessment, particularly in distinguishing peripheral neuropathy from acute inflammatory pathology. Third, incomplete neurological examination during initial assessment, specifically the absence of reflex testing, introduced diagnostic delay, which may not reflect standard clinical practice.

## Conclusions

This case highlights the diagnostic challenges posed by atypical presentations of Guillain-Barré syndrome, particularly when initial symptoms mimic acute cerebrovascular events. It underscores the importance of comprehensive neurological examination, including reflex assessment, and the need to avoid cognitive biases such as diagnostic anchoring. Incorporating structured diagnostic tools such as the Brighton criteria can improve early recognition. Prompt initiation of immunomodulatory therapy and close monitoring for respiratory compromise remain critical to optimising patient outcomes, even in cases with unusual clinical onset.
